# Acute myocardial infarction as the first manifestation of Takayasu arteritis: A case report

**DOI:** 10.1097/MD.0000000000015143

**Published:** 2019-04-12

**Authors:** Ting Zhang, Bo Peng, Xiang Tu, Shan Zhang, Sen Zhong, Wenzhai Cao

**Affiliations:** a School of Clinical Medicine, Chengdu University of Traditional Chinese Medicine, Chengdu; b Department of Nursing, Sichuan Vocational College of Health and Rehabilitation, Zigong; c National Traditional Chinese Medicine Clinical Research Base for Diabetes Mellitus, Affiliated Hospital of Chengdu University of Traditional Chinese Medicine; d School of Nursing, Chengdu University of Traditional Chinese Medicine; e Hospital Management Committee of Chengdu University of Traditional Chinese Medicine, Chengdu; f Department of Cardiology, Zigong First People's Hospital, Zigong, Sichuan, PR China.

**Keywords:** coronary stenosis, myocardial infarction, percutaneous coronary intervention, Takayasu arteritis

## Abstract

**Rationale:**

Takayasu arteritis (TA) is a chronic inflammatory disease involving the aorta and its major branches. Initial diagnosis is usually difficult due to the highly variable symptoms. Acute myocardial infarction (AMI) is a very rare presentation in patients with TA. Moreover, the choice of early management for these patients is not well established.

**Patient concerns:**

A 34-year-old woman was taken to the Emergency Department of our hospital, presenting with a sudden onset and persistent retrosternal chest pain radiating to both upper extremities for 2 hours. Blood pressures were different between 2 arms with 151/115 mm Hg on the right arm and 140/100 mm Hg on the left arm.

**Diagnoses:**

The patient was diagnosed with TA according to the medical history, physical examination, and vascular imaging.

**Interventions:**

Primary percutaneous coronary intervention (PPCI) was performed to restore the coronary flow of left anterior descending. Meanwhile, combination of oral glucocorticoids and immunosuppressive agents was administered to halt disease progression of TA.

**Outcomes:**

Chest pain was relieved without rest and exertional angina. The patient achieved long-term remission without symptom relapse during our follow-up.

**Lessons:**

Percutaneous coronary intervention was essential and effective in AMI of TA. Timely immunosuppressive therapy could improve the long-term outcome.

## 1 Introduction

Takayasu arteritis (TA), now recognized as a worldwide disease, is a well-known yet rare chronic nonspecific inflammatory disease.^[1]^ TA has unknown etiology, predominantly affecting the aorta and its main branches, while small or medium-sized vessels are rarely involved.^[2]^ Nonspecific symptoms could be presented, including upper body pain, intermittent claudication, headache, dizziness, syncope, and visual deterioration. Highly variable vascular lesions could be demonstrated such as arterial stenosis, occlusion, aneurysm, and thrombosis.^[3]^ However, coronary artery involvement manifesting as acute myocardial infarction (AMI) is extremely rare in TA.^[4]^ Furthermore, early reports suggested that the lesions in coronary artery occurred most frequently in ostial sites of right and left main coronary arteries.^[5]^ Our literature describes a rare case of TA initially presenting with AMI and infrequent lesion site of coronary artery. The patient was successfully treated with primary percutaneous coronary intervention, simultaneously initiation of glucocorticoids, and immunosuppressive therapy.

## 2 Case report

The written informed consent was obtained from the patient for publication of this case report and accompanying images. A 34-year-old woman was taken to the Emergency Department of our hospital, presenting with a sudden onset and persistent retrosternal chest pain radiation to both upper extremities pain for 2 hours. The patient denied smoking or drinking habit. She had no known history of hypertension or diabetes, with negative family history for coronary heart disease. Her review of systems was negative.

Her examination revealed blood pressure of 140/100 mm Hg on the left arm and 151/115 mm Hg on the right arm, a heart rate of 120 beats/min, and a temperature of 36.8°C. Rough breathing sounds and moist rale were audible in both lungs. Besides, a scattered, hemorrhagic rash was seen without skin itch. The remainder of the physical examination was unremarkable with negative findings of auscultation of heart or fundus examination.

**Figure 1:**
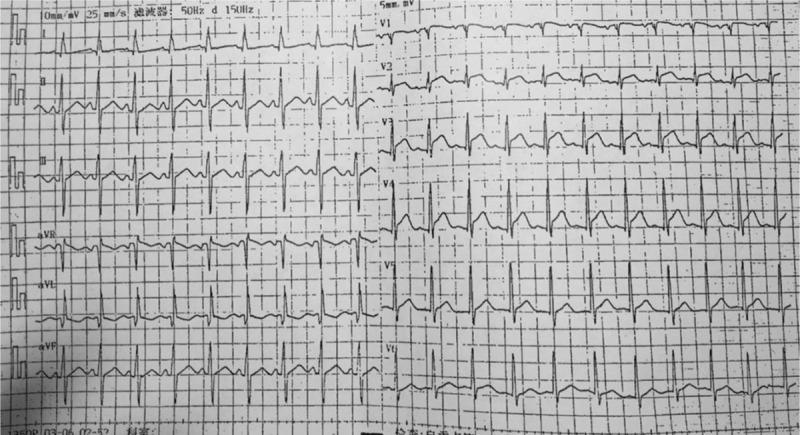
Emergency ECG. ECG demonstrated ST-segment elevation of V1–V5. ECG = electrocardiogram.

An ECG on admission showed sinus tachycardia, ST-segment depression in leads I and aVL, and ST-segment elevation in leads V1 to V5 (Fig. 1) She had high erythrocyte sedimentation rate (ESR, 59 mm/h), elevated C-reactive protein levels (CRP, 29.3 mg/L), and a markedly elevated cardiac troponin I (cTnI, >50 μg/L). Her immunoglobulin levels revealed IgG of 19.1 g/L and IgE of 532 IU/mL, while anti-neutrophil cytoplasmic antibodies (ANCA), C3, and C4 deposits were unremarkable.

**Figure 2:**
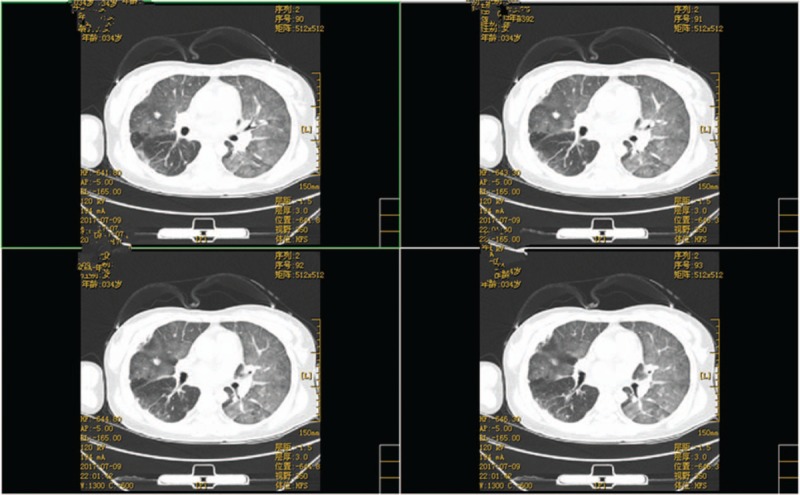
Preoperative CT image. Chest CT revealed pulmonary congestion. CT = computed tomography.

**Figure 3:**
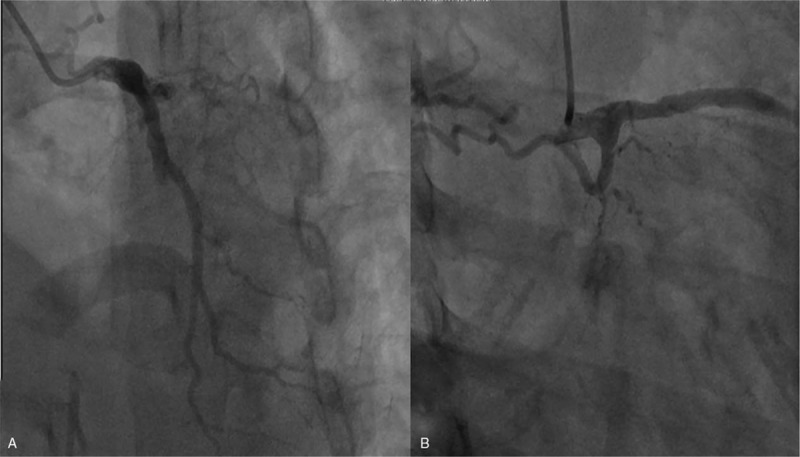
Images of acute coronary angiography. Coronary artery images showed total occlusion in the mid-left anterior descending (A) and mid-left circumflex (B) coronary arteries.

**Figure 4:**
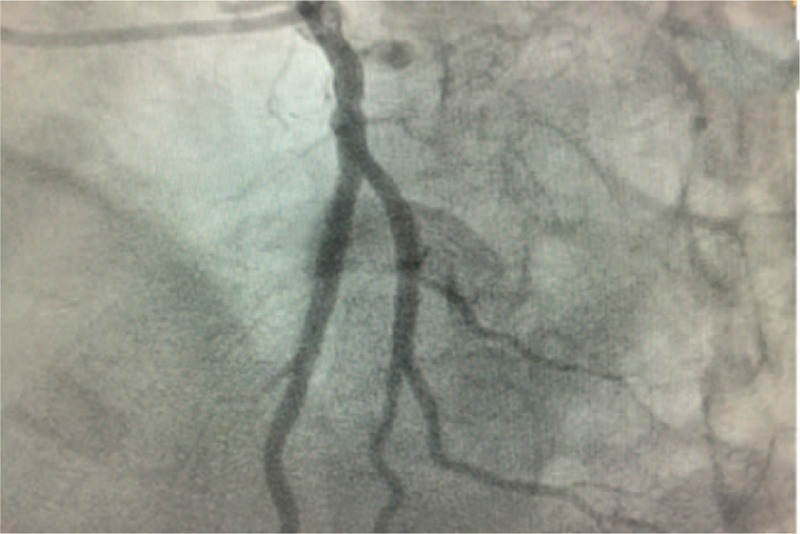
Coronary angiography after stent implantation in anterior descending branch. Coronary angiography revealed that the stent expanded well.

**Figure 5:**
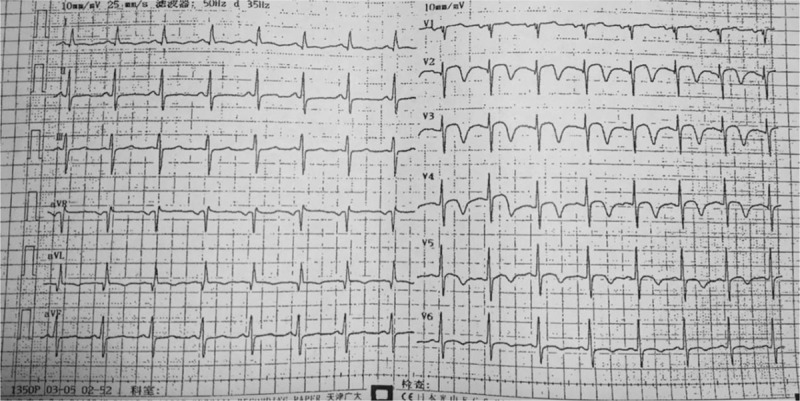
ECG after coronary intervention. ECG revealed decrease in ST segment elevation in the precordial leads and T wave inversion.

High-resolution computed tomography revealed pulmonary edema (Fig. 2). On the basis of the clinical findings, the diagnosis of AMI and pulmonary edema was made immediately. An urgent coronary angiography was initiated, which showed total occlusion in the mid-left anterior descending (LAD) and mid-left circumflex (LCX) coronary arteries (Fig. 3). Therefore, primary percutaneous coronary intervention was performed with implantation of a sirolimus-eluting stent in the mid-LAD artery. Coronary arteriography revealed that the stent expanded well (Fig. 4), normal myocardial perfusion restored with Thrombolysis in Myocardial Infarction (TIMI)-3 flow in the LAD artery. After the procedure, the retrosternal pain was relieved. A later ECG showed a decrease in ST segment elevation in the precordial leads and T wave inversion (Fig. 5).

**Figure 6:**
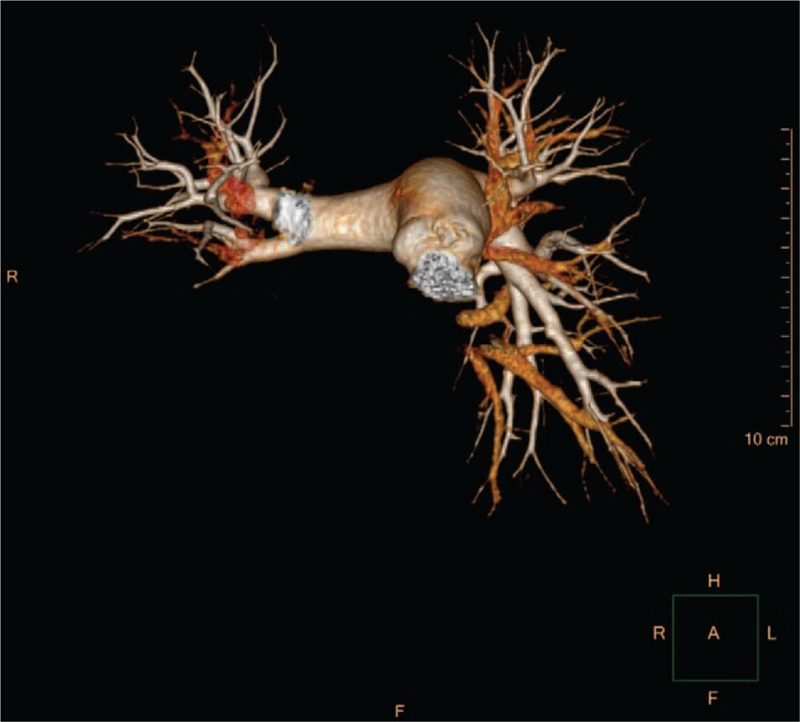
CTA of pulmonary artery. CTA revealed pulmonary artery of right lower lobe was not visualized. CTA = computed tomography angiography.

**Figure 7:**
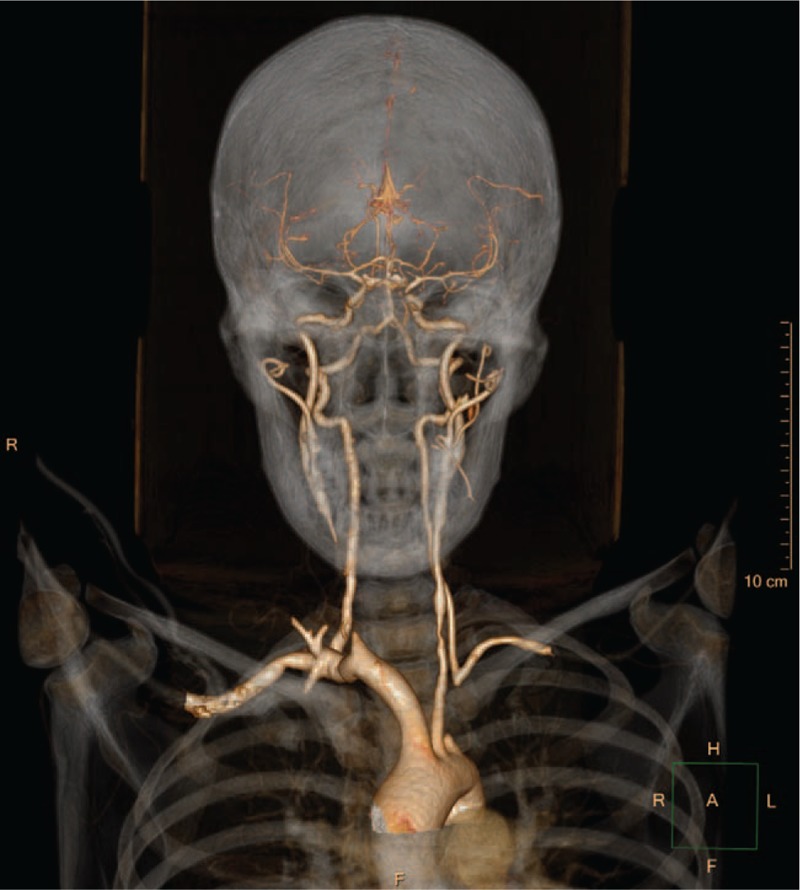
CTA of head and neck artery. CTA showed stenosis in the middle segment of left subclavian artery (LSCA) and mid-distal part of right common carotid artery (RCCA).

**Figure 8:**
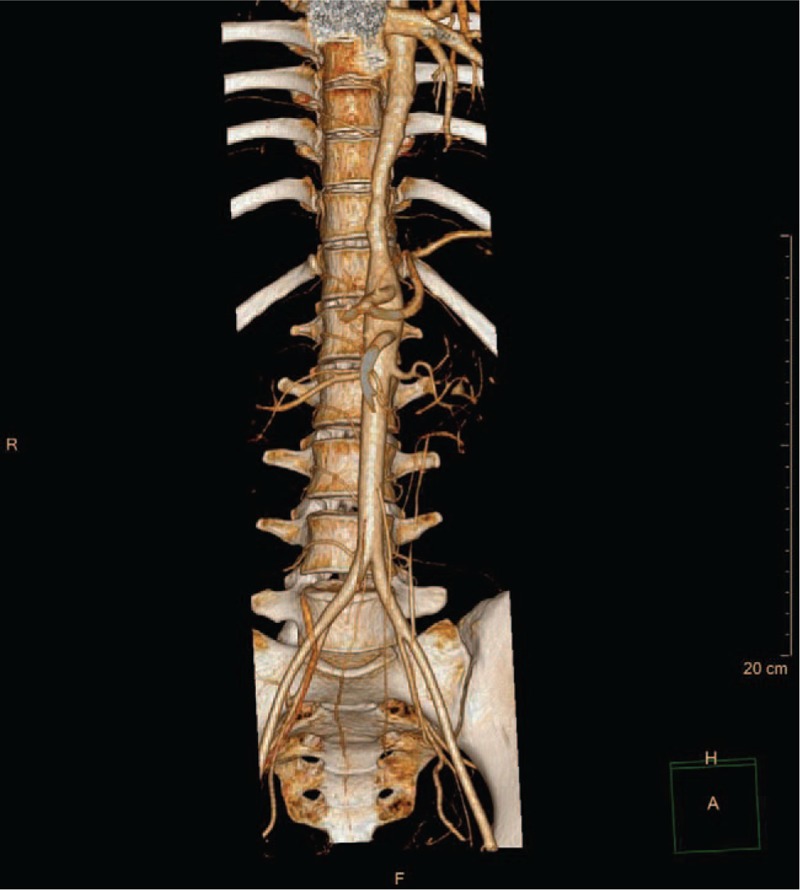
CTA of thoracoabdominal aorta. CTA showed stenosis in the thoracoabdominal aorta.

Further investigation was performed to justify the underlying mechanisms of coronary heart disease in this young patient. Pulmonary computed tomography angiography (CTA) revealed occlusion in right lower lobe artery (Fig. 6). Furthermore, CTA of the aorta demonstrated several arterial lesions, including stenosis in the middle segment of left subclavian artery (LSCA), mid-distal portion of right common carotid artery (RCCA) (Fig. 7), and thoracoabdominal aorta (Fig. 8). On the basis of her medical history, physical examination, and angiographic findings, the patient was determined the diagnosis of TA. The patient was treated with glucocorticoids, immunosuppressive agents, anti-platelet agents, statins with the following regimens: prednisone (2.5 mg/kg qd), methotrexate (10 mg qw), clopidogrel (75 mg qd), aspirin (0.3 g qd), atorvastatin (20 mg qn).

After 12 weeks of treatment, the patient could walk 800 m slowly and climb 3 flights of ordinary stairs at a normal pace without complain of angina. The rash subsided. ESR and CRP decreased within normal range. Echocardiography revealed a slightly larger left ventricular end-diastolic dimension (LVEDD, 54 mm) with hypokinesia for regional wall motion of left inferior posterior ventricle and normal left ventricular ejection fraction (LVEF, 60%).

## 3 Discussion

TA, characterized by massive intimal fibrosis and vascular narrowing, is a rare chronic nonspecific inflammatory disease of undetermined etiology. On the basis of angiographic findings, TA could be classified as type I, II_a_, II_b_, III, IV, or V.^[6]^ The incidence of coronary involvement is relatively low in patients with TA. Diagnostic angiographic and pathologic studies together had revealed coronary artery lesions were in about 9% of TA cases.^[7]^ Despite low incidence, myocardial ischemia is one of the major causes of death in TA, with mortality of up to 50% at 5 years.^[8]^ Therefore, accurate diagnosis and prompt therapy are critical to achievement of complete remission and better survival in TA patients.

In our case, our patient was successfully diagnosed with TA, with the following specific findings.

*Without essential cardiovascular risk factor.* This young woman developed severe coronary heart disease in the absence of common risk factors. We need to be alert for nonatherosclerotic coronary artery disease.

*Markers of inflammation.* ESR and CRP elevation that is disproportionate to coronary heart disease might indicate underlying artery inflammation.

*Gender and blood pressure.* The patient's gender, blood pressure difference between arms made the diagnosis of TA more likely.

*CT angiographic.* The following CT angiographic findings showed that multiple large vessels were involved. According to the classification recommendation of American College of Rheumatology in 1990,^[9]^ the patient who met 3 of 6 criteria (*onset age* *<* *40 years, upper limb BP difference >10* *mm Hg, a*rteriography anomaly) was finally confirmed with TA type V C (+) disease. The relative low incidence, atypical symptoms, and absence of risk factors may lead to misdiagnosis in TA population. To improve the early diagnosis rate of TA, various diagnostic procedures should be evaluated together, including meticulous physical examination, especially the blood pressure of both upper limbs, vascular murmur of neck, upper clavicle or abdominal, laboratory tests such as ESR and CRP, as well as angiographic findings.

Among the previous reports of TA patients with coronary lesions, the ostium of the main coronary artery was most frequently involved, especially the left main stem. The prevalence of ostium lesion was 87.5% as reported by coronary angiography.^[1]^ The ostium of main coronary artery becomes interfered usually due to extension of intimal proliferation and contraction of the fibrotic media and adventitia from the ascending aorta. The mid segment of coronary artery lesion was relatively uncommon in TA patients. However, the inflammatory response could accelerate the development of atherosclerosis directly involving any part even in more distal segments of coronary artery. In our case, the middle segment of LAD and mid-distal part of LCX were affected. Therefore, atypical lesions beyond the ostium of coronary artery should not excluded the possibility of TA.

The treatment of TA includes medications, endovascular intervention, and surgery. During acute inflammatory phase, medical treatment may be initiated with corticosteroids, immunosuppressive agents, anticoagulation and anti-platelet agents, vasodilator drugs, and anti-inflammatory agents, which may effectively suppress vascular inflammation and prompt regression of vessel lesions.^[10]^ Endovascular intervention including percutaneous transluminal angioplasty and stent implantation can be performed only in the remission phase after the patient's condition is stable.^[11]^ As endovascular intervention or surgery could cause damage to the vascular wall, inflammatory response is further accelerated and amplified. Occlusion of the involved vessel or graft, or anastomotic disruption may result in failure of surgery.^[12]^ Mwipatayi et al^[13]^ reported 4 women deaths after surgery in acute inflammatory activity phase. In our case, due to the fatal ischemic symptoms, coronary intervention was still adopted at the acute stage along with the initiation of steroid therapy. Therefore, urgent endovascular intervention could be reasonable in case of stenosis with severe ischemic comorbidity.

Our article presents a rare case of TA with AMI. As relatively uncommon in the patient's presentation, this article entails more research into this field for better diagnosis and management of TA and its complications.

## Author contributions

**Conceptualization:** Ting Zhang, Wen Zhai Cao.

**Data curation:** Bo Peng.

**Project administration:** Wen Zhai Cao.

**Resources:** Sen Zhong.

**Supervision:** Xiang Tu, Sen Zhong.

**Validation:** Shan Zhang, Wen Zhai Cao.

**Writing – original draft:** Ting Zhang.

**Writing – review & editing:** Bo Peng.
